# Cognitive reserve and its impact on cognitive and functional abilities, physical activity and quality of life following a diagnosis of dementia: longitudinal findings from the Improving the experience of Dementia and Enhancing Active Life (IDEAL) study

**DOI:** 10.1093/ageing/afae284

**Published:** 2025-01-07

**Authors:** Laura D Gamble, Linda Clare, Carol Opdebeeck, Anthony Martyr, Roy W Jones, Jennifer M Rusted, Claire Pentecost, Jeanette M Thom, Fiona E Matthews

**Affiliations:** Population Health Sciences Institute, Newcastle University, Newcastle upon Tyne, UK; Centre for Research in Ageing and Cognitive Health, University of Exeter Medical School, Exeter, UK; NIHR Applied Research Collaboration South-West Peninsula, Exeter, UK; Department of Psychology, Manchester Metropolitan University, Manchester, UK; Centre for Research in Ageing and Cognitive Health, University of Exeter Medical School, Exeter, UK; The Research Institute for the Care of Older People, University of Bath, Bath, UK; School of Psychology, University of Sussex, Falmer, UK; Centre for Research in Ageing and Cognitive Health, University of Exeter Medical School, Exeter, UK; School of Health Sciences, The University of Sydney, Sydney, New South Wales, Australia; Institute for Clinical and Applied Health Research, University of Hull, Hull, UK

**Keywords:** leisure activities, Alzheimer’s disease, cognition, education, older people

## Abstract

**Background:**

The concept of cognitive reserve may explain inter-individual differences in susceptibility to neuropathological changes. Studies suggest that experiences over a lifetime impact on cognitive reserve, and it is hypothesised that following a dementia diagnosis, greater reserve levels are linked to accelerated disease progression.

**Objective:**

To investigate the longitudinal impact of cognitive reserve on cognitive and functional abilities, physical activity and quality of life in people with dementia.

**Design:**

Longitudinal cohort design.

**Setting and participants:**

Participants were 1537 people with mild-to-moderate dementia at baseline, 1183 at 12 months follow-up and 851 at 24 months follow-up, from the IDEAL study.

**Methods:**

A comprehensive latent measure of cognitive reserve incorporated domains from all stages of life: education, occupational attainment and later-life engagement in leisure activities. The impact of cognitive reserve on cognition, functional abilities, physical activity and quality of life at baseline and over time was investigated using latent growth curve modelling.

**Results:**

Higher cognitive reserve was associated with better cognition, fewer functional difficulties, more physical activity and better quality of life at baseline but was associated with accelerated cognitive decline and greater dependence over time. After 2 years, those with higher initial reserve were estimated to still have better cognition than those with low reserve.

**Conclusions:**

Cognitive reserve may be important in initially delaying dementia progression but is linked with accelerated deterioration once dementia becomes clinically evident, likely because of the more advanced neuropathological stage of the condition. Engagement in leisure activities is a potentially modifiable domain of cognitive reserve warranting further investigation.

## Key Points

- Higher cognitive reserve was associated with better cognitive and functional ability at baseline.
- Higher cognitive reserve was associated with more physical activity and better quality of life at baseline.
- Higher cognitive reserve was associated with accelerated cognitive decline and greater dependence over time.
- At 2 years follow-up, cognitive function of those with higher cognitive reserve was still better than those with low reserve.
- Engagement in leisure activities may be an avenue for intervention in improving/maintaining cognitive reserve.

## Introduction

Pathological changes related to the development of dementia may start 20 years or more before the clinical presentation of symptoms [1]. Individual differences in the susceptibility to these changes can be attributed to a concept known as cognitive reserve, explaining why some people perform clinically and functionally better despite similar pathology [2, 3]. Research has suggested that lifetime experiences help increase resilience to changes in the brain, contributing to the development of cognitive reserve.

There is sizeable evidence to support a connection between a more active cognitive lifestyle and slower progression of cognitive decline. Studies found that older people were more at risk of developing dementia if they had less education, a low lifetime occupational attainment or engaged in fewer leisure activities [4–9]. Single measures have most commonly been employed, but cognitive reserve is a fluid construct, and a single proxy may not account for the contribution of experiences accumulated across the lifetime. Studies using a composite cognitive lifestyle score, comprising early-life education, mid-life occupation and current social engagement, found that cognitive lifestyle was predictive of decreased dementia incidence over and above other known risk factors [10].

Most studies in this area have explored risk of dementia in healthy older people, with less consideration of the role of cognitive reserve after the point of diagnosis. Studies have shown a more rapid decline and reduced survival time following the onset of clinical symptoms or a diagnosis of dementia in those with higher levels of education, occupational attainment or increased participation in leisure activities [11–16]. Whilst an active cognitive lifestyle is associated with a more favourable trajectory in older people, and low cognitive lifestyle scores with increased risk of cognitive decline, in those with severe impairment high cognitive lifestyle scores were associated with accelerated risk of death [17, 18]. Studies using brain imaging and Alzheimer’s disease biomarkers found that cognitive reserve was initially protective of cognitive decline but resulted in a precipitous decline towards the end stages of Alzheimer’s disease [19–21]. These studies support the theory that higher cognitive reserve can better compensate for initial degenerative brain changes. However, once brain pathology reaches a certain threshold in these people, which varies by individual, clinical symptoms become more evident and the extensive burden of underlying neuropathology leads to a steeper decline in cognition and reduced survival time [3].

There is a need to understand the role of modifiable cognitive reserve markers in maintaining cognitive function in people with dementia. In the present study, we evaluate a comprehensive latent measure of cognitive reserve, incorporating measures from all stages of life, in a large cohort of people living in Britain with a diagnosis of dementia from the Improving the experience of Dementia and Enhancing Active Life (IDEAL) study, and investigate how it affects initial levels of and rate of decline in cognitive function. Since loss of functional ability is associated with cognitive decline [22, 23] and physical activity has been found to improve cognition and functional abilities in people with Alzheimer’s disease [24], we additionally investigate the impact of cognitive reserve on the ability to perform basic and instrumental activities of daily living (ADL) and on engaging in physical activity in people with dementia. Finally, since identifying factors associated with quality of life is a key aim of IDEAL and little research has been conducted on the impact of cognitive reserve on quality of life, we investigate whether cognitive reserve is associated with quality of life.

## Methods

### Design

This study uses longitudinal data from the British IDEAL cohort covering three assessment time points at 12-month intervals [25]. Participants were recruited through 29 UK National Health Service sites throughout Britain between 2014 and 2016. Data were collected through face-to-face interviews in participant’s homes by trained researchers. The IDEAL study was approved by the Wales Research Ethics Committee 5 (reference 13/WA/0405), and the Ethics Committee of the School of Psychology, Bangor University (reference 2014-11684). The IDEAL study was registered with UKCRN, registration number 16593. An involvement group of people with dementia and carers, known as Action on Living Well: Asking You (ALWAYS), assisted with the study design and contributed to interpreting the results [26].

### Participants

Community-dwelling individuals diagnosed with mild-to-moderate dementia [Mini-Mental State Examination (MMSE) [27] score ≥ 15] of any type on enrolment, who were able to provide informed consent, were recruited. Carers providing regular care to the person with dementia contributed if willing to take part [28]. Full criteria for exclusion and consent are provided in the protocol [25]. Our sample comprised 1537 people with dementia and 1266 carers at baseline (Time 1; T1), 1183 people with dementia and 977 carers at 12-month follow-up (Time 2; T2) and 851 people with dementia and 749 carers at 24-month follow-up (Time 3; T3)

### Measures

Supplementary Data Appendix 1 contains additional details on study measures.

#### Domains of cognitive reserve

- Education*—*years of education and level of education (no qualifications, school leaving certificate at 16, school leaving certificate at 18 and university).
- Occupational attainment*—*measures of social class included the Registrar General’s Social Class and the National Statistics Socioeconomic Classification (NS-SEC8) [29, 30].
- Engagement in leisure activities (Supplementary Data Appendix 1; Supplementary Table 1)*—*frequency of participating in twelve cognitive activities [10, 31], and two measures of social activity; the six-item version of the Lubben Social Network Scale [32] and items from the Office for National Statistics social capital survey for the social network and support sub-domain [33, 34].

#### Cognition

Assessed with the Addenbrooke’s Cognitive Examination-III (ACE-III) [35], a brief cognitive test that assesses five cognitive domains: attention, memory, verbal fluency, language and visuospatial ability. The total score (range 0–100) combines these domains. Higher scores indicate better cognitive function. Individual cognitive domains are reported in Supplementary Data Appendix 2.

#### Functional abilities

An 11-item amended version of the Functional Activities Questionnaire (FAQ) measured self-rated and informant-rated ability to perform instrumental ADL (iADL; score range 0–33); higher scores reflect greater impairment [36, 37]. Self-rated and informant-rated FAQ were analysed separately. An informant-rated six-point Dependence Level score was created from questions on the Dependence Scale as described [38]. Scores range from 0 (independent) to 5 (total dependence). Additional measures of functional abilities were investigated, encompassing a range of ADL, and are reported in Supplementary Data Appendix 2.

#### Physical activity

Current physical activity was assessed with the seven-item General Practice Physical Activity Questionnaire (GPPAQ) [39]. A summed total score was used (range 7–29) with higher scores indicating greater activity.

#### Quality of life

The Quality of Life in Alzheimer’s Disease scale (QoL-ad) was used to measure self-rated and informant-rated quality of life [40]. Self-rated and informant-rated QoL-ad were analysed separately. Higher scores indicate better quality of life (score range 13–52).

#### Demographic measures

Type of dementia (determined from medical records), years since diagnosis, age and sex.

### Statistical methods

**Figure 1:**
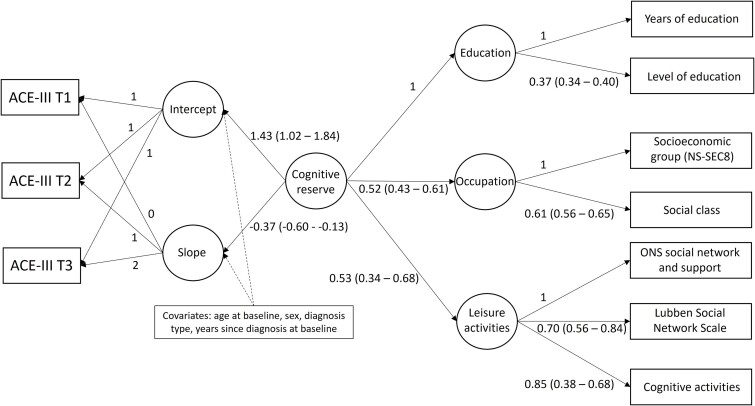
The model diagram for the latent measure of cognitive reserve (right-hand side) and the latent growth curve for cognition (ACE-III total score). Notes: Circles represent latent measures; rectangles represent observed measures. Path estimates are presented alongside 95% CI. Model fit is good: CFI = 0.921, RMSEA = 0.049, SRMR = 0.063.

A latent proxy measure of cognitive reserve was generated in Mplus v8.10 using baseline data and comprised three latent domains: education, occupation and leisure activities. To aid interpretation, all observed measures were coded from low/negative to high/positive; education was coded from fewer years/lower level to more years/higher level, occupational attainment was coded from lower social class/socioeconomic status to higher social class/socioeconomic status, cognitive activities and the ONS social network and support measure were coded from less frequently to more frequently, and the Lubben Social Network Scale was coded from smaller to greater. The model, alongside path coefficients, is shown on the right-hand side of Figure 1. All path coefficients are positive, indicating a positive relationship between the latent and observed measures, and between latent measures.

Descriptive statistics for all study measures were reported. Trajectories of cognition, functional abilities, physical activity and quality of life were investigated using a latent growth curve model (LGCM). The LGCM estimates an intercept (baseline) and slope (rate of change per year), with random effects to account for variation across individuals. The model diagram for cognition is shown in Figure 1. Associations of the latent construct of cognitive reserve or the latent education factor with the intercept and slope of each outcome were investigated. The cognitive reserve latent incorporates aspects of functional abilities in the leisure activities domain that are shared by items in the FAQ. A sensitivity analysis removing these activities from the cognitive reserve latent measure was conducted (Supplementary Data Appendix 1; Supplementary Table 2). Models were adjusted for age at baseline, sex, diagnosis and years since diagnosis at baseline. Full information maximum likelihood estimation was used to handle missing data on outcome measures.

Additional details on the analysis can be found in Supplementary Data Appendix 1.

## Results

### Descriptive statistics

Just over half of participants were male, and ~40% were aged 80 or above (Table 1a). More than half of participants had Alzheimer’s disease (55%) and at baseline, 57% had been diagnosed for <1 year. About 50% of participants had a school leaving certificate at 18 years or above whilst 28% had no qualifications. The mean years of education was 12.5. For occupation, ~45% of participants had a high social class (I/II–professional, managerial and technical roles) and according to the NS-SEC8 ~40% had higher managerial roles (Table 1a). More than 50% of participants listened to the radio or read a newspaper most days, but over half did not play games such as chess and rarely did puzzles (Supplementary Data Appendix 1; Supplementary Table 1A). For social activities (Supplementary Data Appendix 1; Supplementary Table 1b and c), the majority of participants heard from several relatives or friends at least once per month, with most having at least one relative that they could ask for help. Around 70% of participants had a relative they spoke with at least weekly.

**Table 1:** Descriptive statistics for the study cohort.

**a) Person with dementia**			
	**T1 (*N* = 1537)**	**T2 (*N* = 1183)**	**T3 (*N* = 851)**
Sex (*N*, %)			
Male	865 (56.3)	669 (56.6)	476 (55.9)
Female	672 (43.7)	514 (43.4)	375 (44.1)
Age group (*N*, %)			
<65	134 (8.7)	89 (7.5)	66 (7.8)
65–69	177 (11.5)	129 (10.9)	72 (8.5)
70–74	258 (16.8)	193 (16.3)	159 (18.7)
75–79	366 (23.8)	268 (22.7)	172 (20.2)
80+	602 (39.2)	504 (42.6)	382 (44.9)
Dementia type (*N*, %)			
Alzheimer’s disease	851 (55.4)	661 (55.9)	488 (57.3)
Vascular dementia	170 (11.1)	116 (9.8)	82 (9.6)
Mixed Alzheimer’s disease and vascular dementia	324 (21.1)	264 (22.3)	185 (21.7)
Frontotemporal dementia	54 (2.5)	40 (3.4)	32 (3.8)
Parkinson’s disease dementia	44 (2.9)	34 (2.9)	17 (2.0)
Dementia with Lewy bodies	53 (3.4)	39 (3.3)	27 (3.2)
Other/unspecified	41 (2.7)	29 (2.5)	20 (2.4)
Years since diagnosis (*N*, %)			
<1	810 (56.8)	-	-
1–2	448 (31.4)	-	-
3–5	146 (10.2)	-	-
6+	21 (1.5)	-	-
Missing	112		
Years of education (mean, SD, missing)	12.5 (3.7), 45	12.7 (3.5), 28	12.8 (3.5), 20
Level of education (*N*, %)			
No qualifications	429 (28.0)	318 (27.1)	232 (27.4)
Leaving certificate at 16	272 (17.8)	198 (16.9)	136 (16.1)
Leaving certificate at 18	519 (33.9)	411 (35.0)	296 (35.0)
University	311 (20.3)	248 (21.1)	182 (21.5)
Missing	6	8	5
Social class (*N*, %)			
I—Professional	132 (8.8)	103 (9.0)	66 (8.0)
II—Managerial and technical	534 (35.4)	423 (36.8)	316 (38.3)
III-NM—Skilled non-manual	321 (21.3)	234 (20.4)	166 (20.1)
III-M—Skilled manual	313 (20.8)	237 (20.6)	171 (20.7)
IV—Partly skilled	147 (9.8)	109 (9.5)	79 (9.6)
V—Unskilled	39 (2.6)	27 (2.4)	16 (1.9)
Armed forces	21 (1.4)	15 (1.3)	12 (1.5)
Missing/not applicable	30	35	25
NS-SEC8 (*N*, %)			
1—Higher managerial, administrative and professional occupations	240 (15.7)	183 (15.7)	128 (15.2)
2—Lower managerial, administrative and professional occupations	391 (25.6)	307 (26.3)	226 (26.8)
3—Intermediate occupations	263 (17.2)	187 (16.0)	131 (15.5)
4—Small employers and own account workers	185 (12.1)	146 (12.5)	115 (13.6)
5—Lower supervisory and technical occupations	149 (9.8)	119 (10.2)	83 (9.8)
6—Semi-routine occupations	160 (10.5)	124 (10.6)	87 (10.3)
7—Routine occupations	119 (7.8)	82 (7.0)	56 (6.6)
8—Never worked and long-term unemployment	21 (1.4)	20 (1.2)	17 (2.0)
Missing	9	15	8
Cognitive activities (mean, SD, missing)	26.3 (3.7), 64	26.7 (6.8), 51	27.3 (6.9), 37
Lubben social network scale (mean, SD, missing)	15.1 (6.2), 85	15.3 (6.2), 74	15.8 (6.1), 49
ONS social network and support (mean, SD, missing)	12.1 (3.6), 113	14.4 (5.0), 85	14.8 (5.0), 51
Outcome measures (mean, SD, missing)			
Cognition: ACE-III total score	68.6 (13.5), 38	66.4 (15.9), 100	64.6 (17.9), 110
Functional abilities: FAQ score	9.6 (7.7), 54	11.1 (8.4), 181	12.3 (9.0), 113
Physical activity: GPPAQ score	13.4 (3.3), 58	13.1 (3.3), 81	12.9 (3.5), 76
Quality of life: QoL-ad score	36.8 (5.9), 164	37.0 (5.9), 142	37.0 (5.6), 136
Outcome measures (mean, SD, missing)			
Functional abilities: FAQ score	17.9 (8.6), 83	20.8 (8.5), 55	22.4 (8.7), 76
Dependence level	2.79 (1.2), 47	3.06 (1.2), 16	3.34 (1.2), 12
Quality of life: QoL-ad score	33.6 (5.9), 111	32.8 (5.8), 75	31.7 (6.0), 38

Notes: SD, standard deviation; NS-SEC8, National Statistics Socio-economic classification; ACE-III, Addenbrooke’s Cognitive Examination III; FAQ, Functional Activities Questionnaire; GPPAQ, General Practice Physical Activity Questionnaire; QoL-AD, Quality of Life in Alzheimer’s Disease.

For cognition, mean ACE-III total scores decreased by ~2 points between assessment time points. Over time, participants reported a gradual increase in functional difficulties, as reflected by the rise in mean self-rated FAQ scores (1.2–1.5 points per time point). Where carers provided ratings, mean informant-rated FAQ scores were higher than self-rated scores at baseline and increased more between assessment time points (Table 1b). Mean levels of self-rated quality of life remained stable, whereas for those with a participating carer, informant-rated quality of life declined slightly between assessment time points.

### Cognition

**Figure 2:**
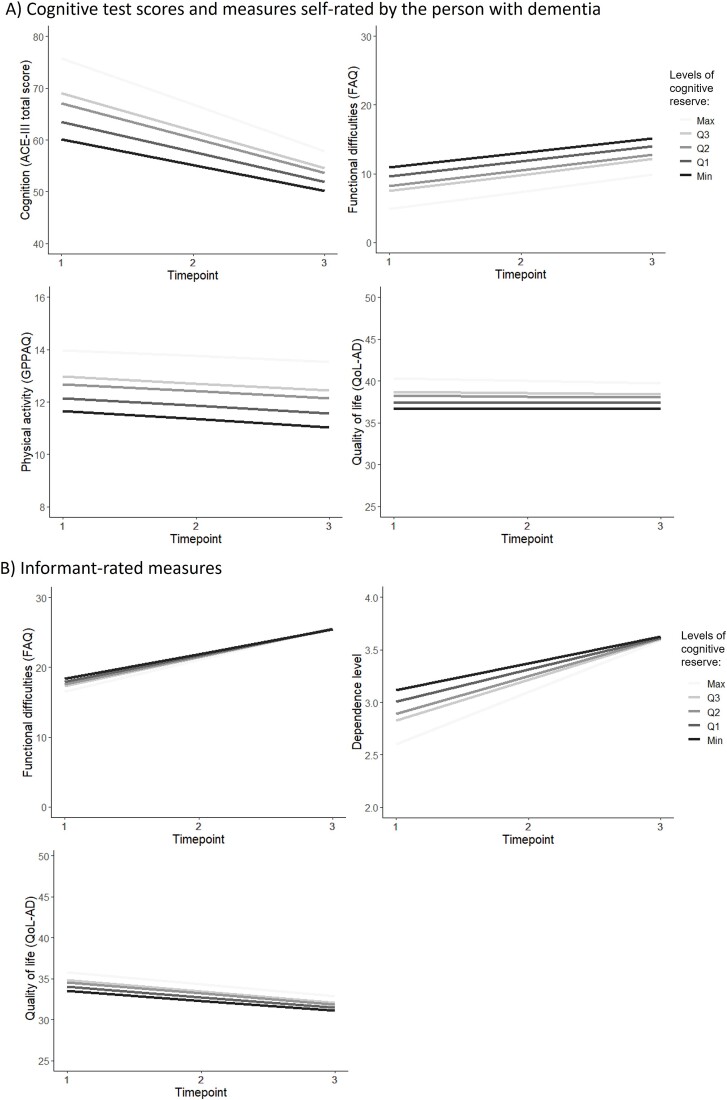
Graphical illustration of the LGCM results presented in Table 2, stratified by varying levels of cognitive reserve. Notes: Graphs are representative of someone who is male, aged 80+ with Alzheimer’s disease, and within the first year of diagnosis at baseline. Levels of the latent measure of cognitive reserve are stratified by the maximum, Q1, Q2 (median), Q3 and the minimum score. For the functional difficulties and dependence level, a higher score indicates more functional difficulties or being more dependent.

The model diagram for investigating the association between cognitive reserve at baseline and rate of change in cognition over time is shown in Figure 1. At baseline a higher cognitive reserve was associated with higher ACE-III total scores, indicating better cognition (Table 2a). However, over time, those with higher cognitive reserve at baseline were estimated to decline at a greater rate, although as shown in Figure 2 they were estimated to still have better cognition at the 2-year follow up (T3) compared to those with lower cognitive reserve.

**Table 2:** Association of a latent measure of cognitive reserve with longitudinal measures of cognition, functional abilities, physical activity and quality of life

**a) Cognitive test scores and measures self-rated by the person with dementia**		
	**Intercept estimate (95% CI)**	**Slope estimate (95% CI)**
Cognition: ACE-III total score	1.43 (1.02–1.84)	−0.37 (−0.60 – −0.13)
Functional abilities: FAQ score	−0.55 (−0.81–−0.30)	0.03 (−0.10–0.17)
Physical activity: GPPAQ score	0.21 (0.12–0.31)	0.01 (−0.05–0.06)
Quality of life: QoL-ad score	0.34 (0.14–0.54)	−0.03 (−0.12–0.06)
**b) Informant-rated measures**		
	**Intercept estimate (95% CI)**	**Slope estimate (95% CI)**
Functional abilities: FAQ score	−0.16 (−0.53–0.20)	0.09 (−0.05–0.22)
Dependence level	−0.05 (−0.08–0.01)	0.02 (0.01–0.04)
Quality of life: QoL-ad score	0.21 (−0.00–0.42)	−0.03 (−0.12–0.07)

Notes: CI, confidence intervals; ACE-III, Addenbrooke’s Cognitive Examination III; FAQ, Functional Activities Questionnaire; GPPAQ, General Practice Physical Activity Questionnaire; QoL-AD, Quality of Life in Alzheimer’s Disease.

For comparison with previous studies mostly looking at education as an indicator of cognitive reserve, the analysis was repeated using the latent education measure, and findings were similar (Table 3). As shown in Figure 1, the latent education factor contributes the most to the cognitive reserve latent measure, but occupational attainment and engagement in leisure activities also impact positively and significantly. Effect sizes for the cognitive reserve latent measure are larger than for the education latent factor alone.

**Table 3:** Association of the latent measure of education with longitudinal measures of cognition, functional abilities, physical activity and quality of life

**a) Cognitive test scores and measures self-rated by the person with dementia**		
	**Intercept estimate (95% CI)**	**Slope estimate (95% CI)**
Cognition: ACE-III total score	1.01 (0.75–1.27)	−0.24 (−0.41—−0.07)
Functional abilities: FAQ score	−0.38 (−0.53 – −0.23)	0.01 (−0.09–0.11)
Physical activity: GPPAQ score	0.16 (0.09–0.22)	0.01 (−0.04–0.05)
Quality of life: QoL-ad score	0.23 (0.11–0.36)	−0.02 (−0.09–0.05)
**b) Informant-rated measures**		
	**Intercept estimate (95% CI)**	**Slope estimate (95% CI)**
Functional abilities: FAQ score	−0.08 (−0.28–0.12)	0.04 (−0.05–0.14)
Dependence level	−0.04 (−0.07—−0.02)	0.02 (0.00–0.04)
Quality of life: QoL-ad score	0.12 (−0.01–0.24)	−0.00 (−0.07–0.07)

### Functional abilities

Whilst higher cognitive reserve at baseline was associated with fewer self-rated functional difficulties in iADL, there was no association between cognitive reserve at baseline and either self-rated or informant-rated ability to perform iADL over time (Table 2; Figure 2). A sensitivity analysis removed overlapping elements of functional ability from the cognitive reserve latent and findings were unchanged (Supplementary Data Appendix 1; Supplementary Table 2). For those with a carer taking part, a higher cognitive reserve at baseline was associated with less informant-rated dependence at baseline but with an increase in dependency over time (Table 2b; Figure 2B). Despite not seeing an association between cognitive reserve and change in functional abilities over time, as shown in Figure 2B the trend over time for informant-rated FAQ was similar to that of dependence when stratified by levels of cognitive reserve; those with greater functional abilities and cognitive reserve at baseline deteriorated at a greater rate. However, given that there was less difference in the range of FAQ scores at baseline according to level of cognitive reserve, the effect over time was less evident.

Again, as for cognition, findings were similar when the analysis was conducted for education only (Table 3).

### Physical activity

Higher cognitive reserve at baseline was associated with increased levels of physical activity at baseline (Table 2a). There was no association between cognitive reserve at baseline and rate of change in physical activity (Figure 2A). Findings were similar for education (Table 3).

### Quality of life

Higher cognitive reserve at baseline was associated with increased self-rated and informant-rated quality of life at baseline (Table 2). However, there was no association between cognitive reserve at baseline and rate of change in quality of life (Figure 2). Findings were similar for education (Table 3).

## Discussion

We found that higher baseline levels of cognitive reserve were linked to higher cognitive function and less dependence compared to those with initial low reserve but were also linked with a greater decline in cognitive function and greater dependence over time. Despite this, people with higher cognitive reserve were still estimated to have better cognitive function after 2-years compared to those with lower reserve. Findings support theories by Stern *et al*., who hypothesised that cognitive reserve accounts for the discontinuity between pathology and clinical outcome; those with higher levels of cognitive reserve tolerate greater neuropathology and are diagnosed at a later stage in the disease, but once dementia is evident the disease is so advanced they show more rapid cognitive decline [3]. Despite the steeper decline, cognitive function would remain higher for those with initial higher reserve, only reaching a similar level at a hypothetical point where memory performance is nil [3].

Many studies show a link between life experiences and dementia risk, but previous studies investigating cognitive reserve in people with Alzheimer’s disease had limitations including using single measures and small sample sizes. We created a comprehensive measure of cognitive reserve, encompassing elements from early life (education), midlife (occupational attainment) and later life (engagement in leisure activities), in >1500 people with a diagnosis of any dementia type. Our study supports and strengthens previous findings [11–15]; higher cognitive reserve results in a more rapid cognitive decline following the onset of clinical symptoms of dementia. Other studies provide further support; more education or higher occupational attainment in people with Alzheimer’s disease, when matched by clinical severity, was linked with poorer survival than those with less [16], and cognitive reserve accelerated cognitive decline, as measured using brain imaging and biomarkers, as dementia progressed [20, 21].

Some studies suggest single measures as proxies of cognitive reserve are sufficient to find a link with cognitive decline [11–15], whilst others looking at dementia risk or mortality show that education needs to be combined with either occupational attainment or leisure activities to see an effect [10, 17, 18]. A meta-analysis found that increased mental activity in late life was associated with lower dementia rates independent of other predictors [41]. We compared findings using the latent cognitive reserve measure to findings using the latent education measure but did not investigate late-life mental activity on its own due to reduced contribution to the latent factor of cognitive reserve in our sample. Results were similar but associations were stronger for the cognitive reserve latent. Each of the three domains of the latent cognitive reserve contributed positively demonstrating the importance of including a comprehensive indicator in future research.

Functional abilities decline at a similar rate as cognitive function in dementia [22, 23] but few studies have investigated the influence of cognitive reserve on functional abilities. One study found that older people with difficulties in performing basic ADL declined at a steeper rate if they had higher initial levels of cognitive reserve, whereas cognitive reserve predicted reduced cognitive decline for older people who had no basic ADL difficulties to begin with [42]. This supports our finding that higher reserve is associated with increasing dependence in people with dementia. No studies have directly investigated the impact of cognitive reserve on the ability to engage in iADL, which is known to rely on cognitive abilities. Surprisingly we did not observe a steeper decline in FAQ scores for those with higher levels of cognitive reserve. However, whilst informant-rated FAQ scores may have a trend towards a steeper decline, there was less difference in the range of scores according to cognitive reserve at baseline meaning less scope to detect a difference.

A single study in healthy older people investigated the relationship between cognitive reserve and quality of life, finding a positive association [43]. Our study extended this finding to people with dementia, suggesting that prolonging cognitive, leisure and/or physical activities may also maintain increased quality of life. The lack of association between cognitive reserve and change in quality of life is unsurprising given that mean quality of life scores are stable over time [44].

Strengths of this study include the large sample and the longitudinal nature of the study. We were able to generate a comprehensive measure of cognitive reserve based on information collected. There were several limitations. As might be expected in a sample of older people with dementia, there was considerable attrition in the cohort, some of which could have been selective. For example, those with lower ACE-III scores may have been more likely to withdraw from the study at the next time point. Only having three time points meant that follow-up time only spanned 2 years, and a linear trend had to be assumed for the LGCM. It would be interesting to see what happens beyond 2 years. At the point of diagnosis, people with higher cognitive reserve show clinical symptoms of dementia, and therefore a steeper decline is expected from this point. We could not investigate decline from the point of diagnosis since people already had dementia when recruited, and instead adjusted for time since diagnosis. Over 50% of participants were diagnosed for less than a year at study entry. Finally, findings may be influenced by another concept known as brain reserve/brain size. For example, more educated individuals may have larger brains or more cortical tissues, protecting them from expressing clinical signs of decline compared to those with low education [7].

Cognitive reserve may be important in promoting better cognitive function, independence, and maintaining quality of life, and in delaying progression of dementia, at least in the early stages of disease pathology. But despite finding that those with higher cognitive reserve deteriorate in their cognitive and functional abilities more than those with lower initial cognitive reserve, the finding that cognition is still better at the 2-year follow up is substantial and means that attempts at maintaining cognitive reserve as the disease progresses are still beneficial. Since all cognitive reserve domains contributed positively to the cognitive reserve measure, positively modifying any one of these could have a positive impact on delaying disease progression. Physical activity also has a positive impact on delaying dementia [45], may itself promote cognitive reserve, and is also important for participation and engagement in leisure and social activities, so is an important aspect to investigate in future research. Since participation in leisure activities is the only domain modifiable in later life, maintaining an active cognitive, social and physical lifestyle may provide an avenue for intervention. However, further studies are needed. Finally, it is likely that those with higher cognitive reserve may need preparations and support systems put in place faster, due to the accelerated deterioration.

## Supplementary Material

aa-24-1075-File004_afae284

## Research data transparency and availability:

IDEAL data were deposited with the UK Data Archive in April 2020. Details of how the data can be accessed can be found here: https://reshare.ukdataservice.ac.uk/854293/.
