# Supplementary material for: Cognitive reserve and its impact on cognitive and functional abilities, physical activity and quality of life following a diagnosis of dementia: longitudinal findings from the Improving the experience of Dementia and Enhancing Active Life (IDEAL) study
Source: Age Ageing. 2025 Jan 7;54(1):afae284. doi: 10.1093/ageing/afae284 (PMC11705083; doi:10.1093/ageing/afae284)
Supplement: aa-24-1075-File004_afae284 [file aa-24-1075-file004_afae284.docx]

**Cognitive reserve and its impact on cognitive and functional abilities, physical activity, and quality of life following a diagnosis of dementia: longitudinal findings from the Improving the experience of Dementia and Enhancing Active Life (IDEAL) study.**

**Supplementary Data**

**Contents**

Appendix 1

- Additional details on study measures.
- Statistical methods.
- Supplementary Table 1. Descriptive statistics for the baseline variables used to calculate the cognitive activities, Lubben Social Network Scale, and Office for National Statistics social network and support sub-domain composite scores that make up the ‘leisure activities’ latent variable.
- Supplementary Table 2. A sensitivity analysis examining the association between the latent measure of cognitive reserve with a modified ‘leisure activities’ component and longitudinal FAQ score.

Appendix 2

- Additional measures of cognition and functional abilities.
- Supplementary Table 3. Descriptive statistics for additional cognition and functional abilities measures.
- Supplementary Table 4. Association of the latent measure of cognitive reserve with longitudinal measures of cognition and functional abilities.
- Supplementary Table 5. Association of the latent measure of education with longitudinal measures of cognition and functional abilities.

References

**Appendix 1**

**Additional details on study measures**

*Occupational attainment*

Measures of social class included the Registrar General’s Social Class and the National Statistics Socioeconomic Classification (NS-SEC) [1, 2]. Both are derived using self-reported information on main occupation and categorised based on the standard occupational classification for the UK [1]. Although both measures are occupation-based, NS-SEC is specifically designed to measure employment relations, i.e., aspects of work and market situations and of the labour contract. While NS-SEC is recommended as a replacement for social class in recent national statistics, the historical Social Class scale focused on measuring the hierarchy of occupations according to their reputed standing within UK society [2]. These two measures are designed to capture different aspects of socioeconomic status. Social Class categories are I - Professional, II - Managerial and technical, III-NM - Skilled non-manual, III-M - Skilled manual, IV - Partly skilled, V – Unskilled, and Armed forces. The NS-SEC8 groups participants into eight classes: 1 - higher managerial, administrative and professional occupations, 2 - lower managerial, administrative and professional occupations, 3 - intermediate occupations, 4 - small employers and own account workers, 5 - lower supervisory and technical occupations, 6 - semi-routine occupations, 7 - routine occupations, 8 - never worked and long-term unemployed.

*Engagement in leisure activities.*

The frequency of participating in 12 cognitive activities was used [3], with scores ranging from 12-60. Examples of cognitive activities included reading a newspaper, doing crossword puzzles and using the internet (see Supplementary Table 1A). Responses were once a year or less, several times a year, several times a month, several times a week, and every day or almost every day. Scores for the Lubben Social Network Scale ranged from 0-30. An example of an item from the Lubben Social Network Scale is ‘How many relatives do you see or hear from once a month’ (see Supplementary Table 1B). Scores for the Office for National Statistics social network and support sub-domain ranged from 0-20, and an example of an item is ‘How often do you speak to friends on the phone’ (see Supplementary Table 1C).

*Cognition*

At T2 and T3 the ACE-III was not administered if the person with dementia scored nine or lower on the MMSE; people with dementia scoring below 10 on the MMSE were administered the Test For Severe Impairment instead [4]. Given that both ACE-III and MMSE measure cognitive function and are strongly correlated (*r* = 0.72 at T1, *r* = 0.83 at T2, and *r* = 0.85 at T3) the MMSE score was used to predict and impute the ACE-III score for these people [5]. In the overall dataset, 25 ACE-III scores were imputed at T2 and 55 were imputed at T3.

*Functional abilities*

Items used to create the Dependence Level score [6] reflecting more extensive disability, such as ‘does the person need to be turned, moved or transferred’, were given more weight. Several additional measures of functional abilities were investigated, encompassing a range of basic and instrumental ADL, and are reported in Supplementary Data “Appendix 2”.

*Physical* *activity*

General Practice Physical Activity Questionnaire (GPPAQ) [7] is a widely used screening tool in general practice surgeries to record the amount of physical activity people engage in.

Quality of life

The Quality of Life in Alzheimer’s Disease scale (QoL-AD) asks participants to rate aspects of their current situation such as physical health, mood, memory, functional abilities, interpersonal relationships, and financial situation, and makes global assessments of satisfaction with self and quality of life [8]. Each item is assessed using a scale from 1 to 4 (poor, fair, good, excellent).

*Diagnosis type*

Diagnosis was recorded as Alzheimer’s disease, vascular dementia, mixed Alzheimer’s disease and vascular dementia, frontotemporal dementia, Parkinson’s disease dementia, dementia with Lewy bodies, or unspecified/other.

*Years since diagnosis*

Years since diagnosis was calculated from date of diagnosis, as taken from medical records, and date of baseline interview.

**Statistical methods**

*Latent measure of cognitive reserve*

A latent proxy variable of cognitive reserve was generated using baseline data. Confirmatory factor analysis is a measurement model that estimates continuous latent variables based on observed indicator variables. First, latent factors of our three domains were generated: 1. Education, 2. Occupation and 3. Leisure activities. The observed variables for education were years of education and level of education. The observed variables for occupation were NS-SEC8 and social class. The final latent factor was leisure activities. For leisure activities, the six-item Lubben Social Network Scale, and composite measures of the twelve cognitive activities items and the nine items from the Office for National Statistics social network were used (see Supplementary Table 1 for the questions used to create these measures). For leisure activities, model fit was better if composite measures for the three components were used rather than the individual items within each component. All observed measures were coded from low/negative to high/positive. Finally, a latent factor of cognitive reserve incorporated the latent measures of education, occupation, and leisure activities.

*Missing data*

Missing data on baseline covariates were imputed using multiple imputation estimated from Markov Chain Monte Carlo simulations. Attrition leads to missing data on outcome measures, and this was handled using full information maximum likelihood (FIML) estimation. FIML estimation adjusts the log-likelihood function to make use of all the complete and partially complete observations and provides valid point estimates and confidence intervals for the parameters of interest. FIML produces unbiased parameter estimates under missing completely at random (MCAR) and missing at random (MAR) missing mechanisms [9].

**Supplementary Table 1. Descriptive statistics for the baseline variables used to calculate the cognitive activities, Lubben Social Network Scale, and Office for National Statistics social network and support sub-domain composite scores that make up the ‘leisure activities’ latent variable**

A) Cognitive activities

|  | How often do you … | | | | | |
| --- | --- | --- | --- | --- | --- | --- |
|  | Listen to the radio | Read the newspaper | Read a magazine | Read a book | Play games such as card or chess | Do crossword puzzles |
|  | N (%) | N (%) | N (%) | N (%) | N (%) | N (%) |
| Once a year or less | 329 (21.4) | 214 (14.0) | 366 (24.0) | 567 (37.1) | 1079 (70.8) | 865 (56.6) |
| Several times a year | 81 (5.3) | 61 (4.0) | 219 (14.4) | 225 (14.7) | 213 (14.0) | 132 (8.6) |
| Several times a month | 124 (8.1) | 187 (12.2) | 415 (27.2) | 157 (10.3) | 112 (7.3) | 134 (8.8) |
| Several times a week | 208 (13.6) | 267 (17.5) | 374 (24.5) | 148 (9.7) | 80 (5.2) | 140 (9.2) |
| Every day or almost every day | 792 (51.6) | 799 (52.3) | 151 (9.9) | 431 (28.2) | 41 (2.7) | 256 (16.8) |
| Missing | 3 | 9 | 12 | 9 | 12 | 10 |

|  | How often do you … | | | | | |
| --- | --- | --- | --- | --- | --- | --- |
|  | Do Sudoku puzzles | Do other types of puzzles | Do jigsaws | Use the internet | Use social media | Play computer games |
|  | N (%) | N (%) | N (%) | N (%) | N (%) | N (%) |
| Once a year or less | 1260 (82.4) | 1059 (69.4) | 1172 (76.9) | 1014 (66.4) | 1398 (91.4) | 1338 (87.2) |
| Several times a year | 51 (3.3) | 117 (7.7) | 215 (14.1) | 52 (3.4) | 11 (0.7) | 29 (1.9) |
| Several times a month | 58 (3.8) | 103 (6.7) | 67 (4.4) | 90 (5.9) | 26 (1.7) | 30 (2.0) |
| Several times a week | 60 (3.9) | 112 (7.3) | 33 (2.2) | 113 (7.4) | 22 (1.4) | 40 (2.6) |
| Every day or almost every day | 100 (6.5) | 136 (8.9) | 38 (2.5) | 259 (17.0) | 72 (4.7) | 97 (6.3) |
| Missing | 8 | 10 | 12 | 9 | 8 | 3 |

B) Social activities: Lubben Social Network Scale items

|  | How many relatives do you see or hear from at least once a month? | How many relatives do you feel at ease with that you can talk about private matters? | How many relatives do you feel close to such that you could call on them for help? | How many of your friends do you see or hear from at least once a month? | How many friends do you feel at ease with that you can talk about private matters? | How many friends do you feel close to such that you could call on them for help? |
| --- | --- | --- | --- | --- | --- | --- |
|  | N (%) | N (%) | N (%) | N (%) | N (%) | N (%) |
| 0 | 60 (4.0) | 123 (8.2) | 73 (4.8) | 240 (16.2) | 513 (34.4) | 361 (24.3) |
| 1 | 100 (6.7) | 261 (17.4) | 187 (12.4) | 157 (10.6) | 209 (14.0) | 198 (13.3) |
| 2 | 204 (13.6) | 316 (21.0) | 306 (20.3) | 180 (12.1) | 254 (17.0) | 266 (17.9) |
| 3 | 233 (15.5) | 256 (17.0) | 254 (16.9) | 146 (9.8) | 140 (9.4) | 165 (11.1) |
| 4 | 229 (15.2) | 210 (14.0) | 233 (15.5) | 164 (11.1) | 134 (9.0) | 137 (9.2) |
| 5 | 167 (11.1) | 115 (7.7) | 144 (9.6) | 120 (8.1) | 61 (4.1) | 90 (6.1) |
| 6 | 127 (8.4) | 80 (5.3) | 110 (7.3) | 127 (8.6) | 62 (4.2) | 92 (6.2) |
| 7 | 76 (5.1) | 26 (1.7) | 46 (3.1) | 21 (1.4) | 11 (0.7) | 12 (0.8) |
| 8 | 73 (4.9) | 3 (2.5) | 60 (4.0) | 59 (4.0) | 21 (1.4) | 30 (2.0) |
| 9 | 44 (2.9) | 11 (0.7) | 15 (1.0) | 17 (1.1) | 9 (0.6) | 11 (0.7) |
| 10 | 66 (4.4) | 30 (2.0) | 30 (2.0) | 86 (5.8) | 37 (2.5) | 50 (3.4) |
| 10+ | 124 (8.3) | 37 (2.5) | 48 (3.2) | 167 (11.3) | 39 (2.6) | 74 (5.0) |
| Missing | 34 | 35 | 31 | 53 | 47 | 51 |

C) Social activities: Office for National Statistics social network and support sub-domain

|  | How often do you speak to relatives on the phone? | How often do you write a letter or note to relatives? | How often do you text or email relatives, or use the internet to talk to relatives? | How often do you speak to friends on the phone? | How often do you write a letter or note to friends? |
| --- | --- | --- | --- | --- | --- |
|  | N (%) | N (%) | N (%) | N (%) | N (%) |
| Never | 108 (7.3) | 1005 (68.0) | 1055 (71.2) | 282 (19.2) | 1062 (71.8) |
| Less often than once a month | 161 (11.0) | 371 (25.1) | 97 (6.5) | 297 (20.3) | 311 (21.0) |
| Once or twice a month | 215 (14.6) | 76 (5.1) | 93 (6.3) | 302 (20.6) | 80 (5.4) |
| Once or twice a week | 571 (38.7) | 19 (1.3) | 148 (10.0) | 436 (29.7) | 24 (1.6) |
| On most days | 421 (28.5) | 7 (0.5) | 89 (6.0) | 149 (10.2) | 3 (0.2) |
| Missing | 61 | 59 | 55 | 71 | 57 |

|  | How often do you text or email friends, or use the internet to talk to friends? | How often do you speak to neighbours? | How often do you meet up with relatives who are not living with you? | How often do you meet up with friends? |
| --- | --- | --- | --- | --- |
|  | N (%) | N (%) | N (%) | N (%) |
| Never | 1144 (77.3) | 51 (3.5) | 62 (4.2) | 177 (12.1) |
| Less often than once a month | 83 (5.6) | 149 (10.1) | 335 (22.8) | 270 (18.4) |
| Once or twice a month | 93 (6.3) | 188 (12.7) | 302 (20.5) | 305 (20.8) |
| Once or twice a week | 98 (6.6) | 470 (31.8) | 450 (36.7) | 574 (39.1) |
| On most days | 61 (4.1) | 618 (41.9) | 231 (15.7) | 142 (9.7) |
| Missing | 58 | 61 | 67 | 69 |

**Supplementary Table 2. A sensitivity analysis examining the association between the latent measure of cognitive reserve with a modified ‘leisure activities’ domain and longitudinal FAQ score**

1. Self-rated FAQ

|  | Intercept  Estimate (95% CI) | Slope  Estimate (95% CI) |
| --- | --- | --- |
| Model 1  Model 2  Model 3 | -0.55 (-0.81 – -0.30)  -0.53 (-0.78 – -0.28)  -0.52 (-0.76 – -0.27) | 0.03 (-0.10 – 0.17)  0.04 (-0.10 – 0.17)  0.05 (-0.09 – 0.20) |

1. Informant-rated FAQ

|  | Intercept  Estimate (95% CI) | Slope  Estimate (95% CI) |
| --- | --- | --- |
| Model 1  Model 2  Model 3 | -0.16 (-0.53 – 0.20)  -0.14 (-0.51 – 0.23)  -0.14 (-0.49 – 0.21) | 0.09 (-0.05 – 0.22)  0.08 (-0.06 – 0.23)  0.10 (-0.05 – 0.25) |

Notes: CI, confidence intervals; FAQ, Functional Activities Questionnaire.

In Model 1, cognitive reserve incorporates all the elements of cognitive reserve as shown in Figure 1. In Model 2, the ‘cognitive activities’ element of the leisure activities domain is excluded. In Model 3, the ‘cognitive activities’ and the ‘ONS social network and support’ element of the leisure activities domain are excluded.

**Appendix 2**

**Additional measures of cognition and functional abilities**

1. Additional methods

1.1 Measures

*Cognition.* Cognition was assessed with the Addenbrooke’s Cognitive Examination-III [10], a brief cognitive test that assesses five cognitive domains: attention (score range 0-18), memory (score range 0-26), verbal fluency (score range 0-14), language (score range 0-26) and visuospatial ability (score range 0-16). For each domain, higher scores indicate better cognitive function.

*Functional abilities.* Several additional measures of functional abilities were included, encompassing basic ADL and instrumental ADL. For basic ADL, these were self-rated single questions relating to self-care (I have no problems with self-care, I have some problems with self-care, and I am unable to wash or dress myself), and performing usual activities (I have no problems performing my usual activities, I have some problems with performing my usual activities and I am unable to perform my usual activities) [11]. Both were coded into binary measures; ‘I have problems with self-care/performing usual activities’ and ‘I do not have problems with self-care/performing usual activities’. Some informant-rated measures were also used to create a measure ‘does not need help with basic ADL’ and ‘needs help with basic ADL’; the person with dementia was considered to need help with basic ADL if the informant answered yes to any of the following questions taken from the Dependence Scale [6]: Does your relative/friend need to be accompanied when bathing or eating? Does your relative/friend have to be dressed, washed, and groomed? Does your friend have to be taken to the toilet regularly to avoid incontinence? Does your friend/relative have to be fed? Does your relative/friend have to be turned, moved or transferred? Does your friend/relative wear an incontinence pad or catheter? Does your friend/relative have to be tube fed? Finally, the eleven-item amended version of the Functional Activities Questionnaire (FAQ), used to measure self-rated and informant-rated ability to perform instrumental ADL (score range 0-33), was split into the two widely used cut-off scores for impairment found in the literature, where scores >5 or >9 indicate impairment [12, 13]. Because the distribution of the total score for continuous FAQ is skewed and this may impact on the results, these binary measures were used to confirm the findings.

*1.2 Latent growth curve model for binary measures*

For binary outcomes, a logit link was specified with thresholds held constant over time for model identification purposes [14].

*2. Findings*

*2.1 Descriptives*

Descriptive statistics of the additional study measures are found in Supplementary Table 3. Mean ACE-III scores of each of the five subdomains decreased slightly from T1 to T3. In terms of basic ADL, almost 20% of people with dementia indicated that they needed help with self-care, and approximately 34% indicated that they had problems doing their usual activities at baseline. For people with dementia who had a carer taking part (N = 1266 at T1), at baseline 27% of carers indicated that the person they were caring for needed help with basic ADL. All measures of basic ADL worsened with time.

The FAQ was used as a measure of ability to conduct instrumental ADL. Both used cut points (>5 and >9) for ‘functional impairment’ for this measure were reported; 63% self-reported as having functional impairment with the lower cut off of >5, and 43% with the higher cut-off of >9. Ratings of functional impairment by the carer were much higher, with 90% reporting functional impairment with the lower cut off of >5, and 81% with the higher cut point of >9. The proportion rated as functionally impaired increased with time.

*2.2 Model results*

Over time, the ACE-III subdomain scores for attention, verbal fluency and memory of those with a higher cognitive reserve at baseline declined at a greater rate (Supplementary Table 4). There was an association between cognitive reserve and the language and visuospatial ACE-III subdomains at baseline, but there was less certainty about a relationship over time.

Those with higher cognitive reserve at baseline needed less help with self-care and were more able to do their usual activities. For those with a carer taking part, the carer was less likely to rate the person with dementia as needing help with basic ADL if they had higher cognitive reserve. There were no associations with cognitive reserve and change in self-rated basic ADL measures over time. However, for the informant-rated measure of needing help with basic ADL, those with higher initial cognitive reserve needed more help with basic ADL over time.

For the binary measures of functional impairment, again, despite seeing a difference at baseline, with higher cognitive reserve associated with less self-rated functional impairment, there were no associations between cognitive reserve and change over time. The association between informant-rated functional impairment and cognitive reserve was less clear.

Results were similar for the latent measure of education (Supplementary Table 5).

**Supplementary Table 3. Descriptive statistics for additional cognition and functional abilities measures.**

1. Cognitive test scores and measures self-rated by the person with dementia

|  | T1 (N = 1537) | T2 (N = 1183) | T3 (N = 851) |
| --- | --- | --- | --- |
| *Cognition* |  |  |  |
| ACE-III attention  (mean (sd), missing) | 13.9 (3.0), N=31 | 13.0 (3.5), N=88 | 12.6 (3.8), N=107 |
| ACE-III verbal fluency  (mean (sd), missing) | 6.8 (3.1), N=32 | 6.4 (3.2), N=91 | 6.2 (3.4), N=108 |
| ACE-III language  (mean (sd), missing) | 21.8 (3.8), N=38 | 21.9 (4.3), N=100 | 21.2 (5.1), N=110 |
| ACE-III memory  (mean (sd), missing) | 13.6 (5.4), N=38 | 12.9 (6.0), N=96 | 12.7 (6.3), N=110 |
| ACE- III visuospatial  (mean (sd), missing) | 12.5 (3.3), N=38 | 12.2 (3.5), N=100 | 11.8 (3.8), N=110 |
| *Functional abilities* |  |  |  |
| Self-care (N, %): |  |  |  |
| I have no problems with self-care | 1266 (82.5) | 929 (79.3) | 654 (78.5) |
| I have problems with self-care | 269 (17.5) | 242 (20.7) | 179 (21.5) |
| Missing | 2 | 12 | 18 |
| Usual activities (N, %): |  |  |  |
| I have no problems performing my usual activities | 1015 (66.3) | 751 (64.2) | 522 (62.7) |
| I have problems performing my usual activities | 515 (33.7) | 419 (35.8) | 310 (37.3) |
| Missing | 7 | 13 | 19 |
| FAQ: functional impairment (N, %): |  |  |  |
| 0-5 (below cut score) | 552 (37.2) | 310 (30.9) | 217 (29.4) |
| 6-33 (cut score and above) | 931 (62.8) | 692 (69.1) | 521 (70.6) |
| 0-9 (below cut score) | 839 (56.6) | 490 (48.9) | 322 (43.6) |
| 10-33 (cut score and above) | 644 (43.4) | 512 (51.1) | 416 (56.4) |
| Missing | 54 | 181 | 113 |

B) Informant-rated measures

|  | T1 (N = 1266) | T2 (N = 977) | T3 (N = 749) |
| --- | --- | --- | --- |
| *Functional abilities* |  |  |  |
| FAQ: functional impairment (N, %): |  |  |  |
| 0-5 (below cut score) | 122 (10.4) | 65 (7.0) | 36 (5.0) |
| 6-33 (cut score and above) | 1050 (89.6) | 869 (93.0) | 688 (95.0) |
| 0-9 (below cut score) | 219 (18.7) | 110 (11.8) | 75 (10.4) |
| 10-33 (cut score and above) | 953 (81.3) | 824 (88.2) | 649 (89.6) |
| Missing | 94 | 43 | 25 |
| Help with basic ADL (N, %): |  |  |  |
| Does not need help | 896 (73.1) | 608 (63.1) | 388 (53.0) |
| Needs help | 328 (26.9) | 352 (36.9) | 341 (47.0) |
| Missing | 42 | 17 | 20 |

Notes: sd, standard deviation; ACE-III, Addenbrook’s Cognitive Examination III; FAQ, Functional Activities Questionnaire; ADL, activities of daily living.

**Supplementary Table 4. Association of the latent measure of cognitive reserve with longitudinal measures of cognition and functional abilities.**

1. Person with dementia self-completed measures

|  | Intercept | Slope |
| --- | --- | --- |
| *Cognition* | Estimate (95% CI) | Estimate (95% CI) |
| ACE-III attention | 0.24 (0.15 – 0.33) | -0.06 (-0.12 – -0.01) |
| ACE-III verbal fluency | 0.20 (0.12 – 0.29) | -0.07 (-0.11 – -0.03) |
| ACE-III language | 0.36 (0.25 – 0.48) | -0.06 (-0.12 – 0.01) |
| ACE-III memory | 0.36 (0.20 – 0.52) | -0.09 (-0.17 – -0.01) |
| ACE- III visuospatial | 0.19 (0.10 – 0.28) | -0.03 (-0.08 – 0.01) |
| *Functional abilities* | OR (95% CI) | OR (95% CI) |
| Needs help with self-care | 0.82 (0.70 – 0.97) | 1.07 (0.97 – 1.19) |
| Cannot perform usual activities | 0.89 (0.80 – 0.99) | 1.03 (0.97 – 1.11) |
| FAQ: impaired (6-33) | 0.77 (0.68 – 0.87) | 0.99 (0.90 – 1.10) |
| FAQ: impaired (10-33) | 0.75 (0.65 – 0.87) | 1.00 (0.91 – 1.10) |

1. Informant-rated measures

|  | Intercept | Slope |
| --- | --- | --- |
| *Functional abilities* | OR (95% CI) | OR (95% CI) |
| FAQ: impaired (6-33) | 0.63 (0.51 – 0.94) | 1.17 (0.98 – 1.40) |
| FAQ: impaired (10-33) | 0.86 (0.66 – 1.11) | 1.08 (0.62 – 1.27) |
| Needs help with basic ADL | 0.74 (0.60 – 0.91) | 1.14 (1.03 – 1.26) |

Notes: CI, confidence intervals; OR, odds ratio; ACE-III, Addenbrooke’s Cognitive Examination III; FAQ, Functional Activities Questionnaire; ADL, activities of daily living.

**Supplementary Table 5. Association of the latent measure of education with longitudinal measures of cognition and functional abilities.**

1. Person with dementia self-completed measures

|  | Intercept | Slope |
| --- | --- | --- |
| *Cognition* | Estimate (95% CI) | Estimate (95% CI) |
| ACE-III attention | 0.16 (0.11 – 0.22) | -0.04 (-0.08 – 0.00) |
| ACE-III verbal fluency | 0.17 (0.11 – 0.23) | -0.06 (-0.10 – -0.03) |
| ACE-III language | 0.27 (0.20 – 0.34) | -0.04 (-0.09 – 0.01) |
| ACE-III memory | 0.28 (0.17 – 0.39) | -0.07 (-0.13 – -0.00) |
| ACE- III visuospatial | 0.15 (0.09 – 0.20) | -0.02 (-0.06 – 0.02) |
| *Functional abilities* | OR (95% CI) | OR (95% CI) |
| Needs help with self-care | 0.87 (0.78 – 0.97) | 1.06 (0.98 – 1.15) |
| Cannot perform usual activities | 0.91 (0.84 – 0.99) | 1.03 (0.97 – 1.09) |
| FAQ: impaired (6-33) | 0.83 (0.76 – 0.90) | 0.99 (0.92 – 1.07) |
| FAQ: impaired (10-33) | 0.81 (0.74 – 0.89) | 1.00 (0.93 – 1.07) |

1. Informant-rated measures

|  | Intercept | Slope |
| --- | --- | --- |
| *Functional abilities* | OR (95% CI) | OR (95% CI) |
| FAQ: impaired (6-33) | 0.75 (0.60 – 0.95) | 1.13 (0.96 – 1.33) |
| FAQ: impaired (10-33) | 0.89 (0.68 – 1.15) | 1.08 (0.92 – 1.28) |
|  | OR (95% CI) | OR (95% CI) |
| Needs help with basic ADL | 0.77 (0.65 – 0.90) | 1.12 (1.02 – 1.23) |

Notes: CI, confidence intervals; OR, odds ratio; ACE-III, Addenbrooke’s Cognitive Examination III; FAQ, Functional Activities Questionnaire; ADL, activities of daily living.

**References**

1. Office for National Statistics. The national statistics socioeconomic classification: (Rebased on the SOC2010) User Manual. Basingstoke: 2010.

2. Rose D. Official Social Classifications in the UK. UK: Department of Sociology, University of Surrey; 1995; Available from: <https://sru.soc.surrey.ac.uk/SRU9.html>.

3. Valenzuela M, Brayne C, Sachdev P et al. Cognitive lifestyle and long-term risk of dementia and survival after diagnosis in a multicenter population-based cohort. *Am J Epidemiol*. 2011; 173(9): 1004-12. doi: 10.1093/aje/kwq476.

4. Albert M, Cohen C. The Test for Severe Impairment: an instrument for the assessment of patients with severe cognitive dysfunction. *J Am Geriatr Soc*. 1992; 40(5): 449-53. doi: 10.1111/j.1532-5415.1992.tb02009.x.

5. Martyr A, Ravi M, Gamble LD et al. Trajectories of cognitive and perceived functional decline in people with dementia: Findings from the IDEAL programme. *Alzheimers Dement*. 2023; 20(1): 410-20. doi: 10.1002/alz.13448.

6. Stern Y, Albert SM, Sano M et al. Assessing patient dependence in Alzheimer's disease. *J Gerontol*. 1994; 49(5): M216-22. doi: 10.1093/geronj/49.5.m216.

7. National Health Service. The General Practice Physical Activity Questionnaire (GPPAQ): a screening tool to assess adult physical activity levels, within primary care. London: Department of Health; 2009.

8. Logsdon RG, Gibbons LE, McCurry SM et al. Quality of life in Alzheimer's disease: patient and caregiver reports. In: Albert SM, Logsdon RG, editors. Assessing quality of life in dementia. New York: Springer; 2000. p. 17-30.

9. Cai J, Zeng D, Li H et al. Comparisons of statistical methods for handling attrition in a follow-up visit with complex survey sampling. *Stat Med*. 2023; 42(11): 1641-68. doi: 10.1002/sim.9692.

10. Hsieh S, Schubert S, Hoon C et al. Validation of the Addenbrooke's Cognitive Examination III in frontotemporal dementia and Alzheimer's disease. *Dement Geriatr Cogn Disord*. 2013; 36(3-4): 242-50. doi: 10.1159/000351671.

11. EuroQol G. EuroQol--a new facility for the measurement of health-related quality of life. *Health Policy*. 1990; 16(3): 199-208. doi: 10.1016/0168-8510(90)90421-9.

12. Martyr A, Nelis SM, Quinn C et al. The relationship between perceived functional difficulties and the ability to live well with mild-to-moderate dementia: Findings from the IDEAL programme. *Int J Geriatr Psychiatry*. 2019; 34(8): 1251-61. doi: 10.1002/gps.5128.

13. Pfeffer RI, Kurosaki TT, Harrah CH, Jr. et al. Measurement of functional activities in older adults in the community. *J Gerontol*. 1982; 37(3): 323-29. doi: 10.1093/geronj/37.3.323.

14. Lee TK, Wickrama K, O'Neal CW. Application of latent growth curve analysis with categorical responses in social behavioral research. *Struct Equ Modeling*. 2018; 25(2): 294-306. doi: 10.1080/10705511.2017.1375858.
